# Optimization and Its Implementation Impact of Two-Modes Controller Fractional Approximation for Buck Converters

**DOI:** 10.3390/mi13101600

**Published:** 2022-09-26

**Authors:** Allan G. S. Sánchez, Francisco-Javier Perez-Pinal, Alejandro Espinosa-Calderón

**Affiliations:** 1 CONACYT-Tecnológico Nacional de México en Celaya, Celaya 38010, Mexico; 2 Tecnológico Nacional de México en Celaya, Celaya 38010, Mexico; 3 Regional Center for Optimization and Device Development (CRODE), Tecnológico Nacional de México, Celaya 38020, Mexico

**Keywords:** fractional control, DC-DC converters, optimization algorithms, particle swarm algorithm, fractional-order implementation

## Abstract

Additional degrees of freedom in a fractional-order control strategy for power electronic converters are well received despite the lack of reliable tuning methods. Despite artificial/swarm intelligence techniques have been used to adjust controller parameters to improve more than one characteristic/property at the same time, smart tuning not always leads to realizable structures or reachable parameter values. Thus, adjustment boundaries to ensure controller viability are needed. In this manuscript the fractional-order approach is described in terms of El-Khazali biquadratic module, which produces the lowest order approximation, instead of using a definition. A two-modes controller structure is synthesize depending on uncontrolled plant needs and parameters are adjusted through particle swarm and genetic optimization algorithms for comparison. Two error-based minimization criteria are used to consider output performance into the process. Two restrictions complement the optimization scheme, one seeks to ensure desired robustness while the other prevents from synthesizing a high-gain controller. Optimization results showed similarity between minima obtained and significant difference between parameters of those controller optimized without the proposed constraints was determined. Numerical and experimental results are provide to validate proposed approach effectiveness. Effective regulation, good tracking characteristic and robustness in the presence of load variations are the main results.

## 1. Introduction

Power electronic converters (PECs) main purpose is to modify power signal characteristics to those required by a specific machine or device. Direct current to direct current (DC-DC) conversion is one of the most relevant functionalities of PECs, since they are not only used in low/high power industrial and household applications, but also employed in biomedical devices for health care through diagnosis, treatment and rehabilitation.

DC-DC buck converter plays a key role in biomedical devices, whose power subsystems have to perform energy harvesting, storage, and management tasks efficiently within a limited space, commonly at micro/nano scale. Pacemakers, defibrillators, cochlear processors, retinal stimulators, neural recording and body-area monitoring are on-chip devices with highly limited energy sources, which are deeply benefited by the improvement of existing techniques and alternative proposal for controlling PECs [1,2,3,4]. Some other application of DC-DC buck converter include battery charging [5], renewable energy conversion systems [6], microgrids [7], regulated power sources [8], LED lightning [9] and DC motor drives [10].

In the last decade, a popular line of action to achieve voltage regulation on PECs is to consider a known control strategy that is adapted to integrate fractional calculus to its description in combination with techniques such as Artificial Neural Networks (ANN), Fuzzy Logic (FL) or Deep Learning (DL) from artificial intelligence or optimization algorithms (OA) to be used as tuning method. This approach seeks to improve controller performance through the appropriate choice of its parameters. Bio-inspired optimization algorithms such as Particle Swarm (PSOA), Whale (WOA), Bat (BOA), Black Widow (BWOA), Evolutionary (EOA) or Genetic (GOA) are the most commonly used due to their simplicity and physical interpretation.

Fractional calculus integration into control of dynamic systems has been well received due to the theoretical development that made its interpretation, approximation and realization possible. The PECs field has benefited from the fractional-order control (FOC) development, which derived in more accurate and flexible/robust strategies [11,12] or more precise controllers [13,14].

Some resent and relevant results on FOC applied to PECs can be summarized as follows: highly sophisticated proposals that included either fuzzy- or MPPT-based PID controllers were suggested in [15,16] to deal with disturbances and uncertainties when regulating output voltage in a buck converter. In addition, controller parameters tuning was performed through optimization algorithms. The combination resulted in effective regulation and suppression of instability effects caused by a constant power load of microgrids. In [17] an anti windup controller based on the fractional PI structure was investigated. The approach was proposed to control a motor through a buck converter for low speed applications. Benefits of the proposal were a softer switching pulse, superior tracking speed, steady-state reduction and wind up phenomena removal. In [18], a fractional PI controller was proposed to regulate output voltage in a buck converter. The non-integer structure was used to eliminate steady-state error and oscillations. Response rate convergence was enhanced by cascading the fractional PI with a compensator of fuzzy-logic nature. A PSOA was used to determine parameters of the combined control strategy. Minimum error and improved disturbance-rejection capability were the main results.

In [19], artificial ecosystem optimization algorithm and Nelder-Mead simplex method were combined to optimize parameter values of a fractional-order PID structure to regulate output voltage in a buck converter. Error-based minimization criteria were proposed to ensure output performance. Transient and frequency response enhancement were the main benefits from the approach. In [20], the improved hunger games search optimization algorithm was used to determine fractional PID parameters to achieve voltage regulation in a buck converter. Oustaloup recursive approximation was suggested to achieve controller implementation. Enhanced transient, robustness against load variations, uncertainties and noise were the most relevant results produced by the proposal. In [21], a general structure of fractional-order PID controller was suggested to regulate output voltage in a buck converter. Controller parameters were chosen in an optimization scheme that included disturbance rejection and robustness criteria that were maximized by combining Lévy flight distribution and simulated annealing algorithms. Short transient, good disturbance rejection and a better closed-loop response were the improvements.

A fractional sliding and fractional-terminal sliding mode controllers with back-stepping and reset control for regulating voltage in a buck converter were proposed in [22,23]. Designed controllers considered uncertainties rejection, non-linear loads and non-modeled dynamics. Steady-state error reduction, perturbation rejection enhancement and $L_{2}$-stability in Lyapunov sense were the main results. A backstepping-based control, in combination with the ANN technique to estimate controller parameters, was investigated in [24]. Constant and stable voltage supply and good disturbance rejection were the improvements.

In [25], the common fractional-order pole/zero placement through k-factor-based approach was investigated to control the buck converter output. By using a classical structure of compensator to represent the controller, stability region can be extended or simply guaranteed by establishing phase margin. The main improvement was robustness against load variations and parametric uncertainties.

In a different approach but considering the fractional representation of the system to be controlled, Refs. [26,27,28] proposed the Caputo or Grünwald-Letnikov fractional definitions to integrate the non-integer representation of a system into the control scheme. As was expected, the controller design, which was called the constrained approach, have to considered additional restrictions to ensure basic properties such as stability in the sense of Lyapunov and controllability. By defining either a state feedback or a predictive control scheme effective convergence to the desired value and stability were corroborated.

Two paths can be distinguished from the way in which the above described works addressed the non-integer order approach. On one hand, by using a fractional-order definitions to describe derivatives/integrals, among which one can find Riemann-Liouville, Caputo, Grünwald-Letnikov, Liouville, Weyl, Marchaud, Hadamard, Chen and Atangana-Baleanu [29], to mention the most relevant, being Riemann-Liouville, Caputo and Grünwald-Letnikov the most employed. On the other hand, an alternative way is to approximate the Laplacian operator through integer-order transfer functions. Some of the most used approximation techniques are Carlson [30], Oustaloup [31], refined Oustaloup [32], Charef [33] and El-Khazali [34,35], among others. Industry and applied researchers main concern is the way fractional-order models are implemented. Non-integer order approximation through high order transfer function might represent a viable option that would lead to realizable models.

Physical implementation of fractional-order PI/PD/PID controllers can be achieved by approximating its structure through rational functions of polynomials, whose partial fraction expansion can be generated with a three- to five-term electrical arrangement by using RC networks and operational amplifiers.

In this manuscript optimization impact over the implementation viability of a fractional-order approximation of two-modes controller is analyzed. Bio-inspired optimization algorithms will be used to determine controller parameters. Two different error-based minimization criteria are used to consider performance of system’s output into the process. To complete the optimization scheme, robustness and controller implementation viability were integrated through two relevant optimization restrictions, which consider acceptable closed-loop phase margin and limits the controller phase contribution to what is necessary to reach it, thus avoiding the synthesis of high-gain controllers. Controller structure will be chosen according to system’s needs by combining proportional mode with either derivative or integral one to achieve voltage regulation at the plant’s output. Effectiveness of resulting controller is validated numerically and experimentally.

The manuscript is organized as follows: in Section 2 necessary preliminaries on the three topics that are included in this work are described. Brief review on DC-DC buck converter operation and its model are provided in this section. The biquadratic module to approximate the controller fractional order and the optimization scheme are explained in this section as well. Results of the optimization process and numerical simulation of the electrical behavior predicting its impact on the controller realization are explained in Section 3. Experimental validation corroborating effectiveness of the proposed approach is provided also in this section. Some discussion on the relevance and effectiveness of obtained results are provided in Section 4. Lastly, conclusion on the results presented and some future directions of this work are provided in Section 5

## 2. Materials and Methods

In this manuscript three major topics are combined. Firstly, DC-DC converters from which the buck configuration is chosen as the dynamical system to be controlled. Studying this conversion topology is important due to its vast range of applications, which includes industrial, household and biomedical ones.

Secondly, fractional-order control has become relevant since the appearance of methods for solving fractional-order equations. In the last 30 years many control strategies have been proposed or modified to integrate the fractional-order approach resulting in significant performance improvement.

Lastly, optimization represents the main resource to achieve control objectives efficiently by setting the appropriate controller parameter values. Fractional-order control has mainly resorted to meta-heuristic optimization algorithms to searching and testing potential solutions as alternative to improve controller performance.

In the following, a brief review on these topics is provided for a better understanding of the manuscript.

### 2.1. DC-DC Buck Converter

**Figure 1:**
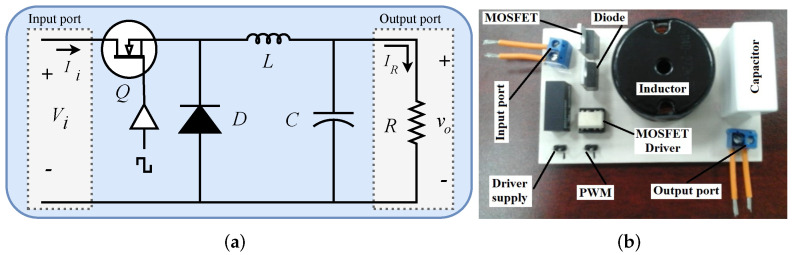
Buck converter. (**a**) Electrical diagram. (**b**) Electrical implementation.

Buck converter is one of the most used configuration from DC-DC conversion. It is characterized by the ability of stepping down its source of power, this means that at the output a controlled level of lower voltage is provided to the load *R*. Stepping down converter’s power supply $V_{i}$ can be achieved by using a capacitor *C*, an inductor *L*, a diode *D* and a MOSFET *Q* connected as shown in Figure 1, which shows the electrical diagram of buck converter and its physical implementation. Parameter specifications and generals for components and elements of buck converter shown in Figure 1 are listed in Table 1.

**Figure 2:**
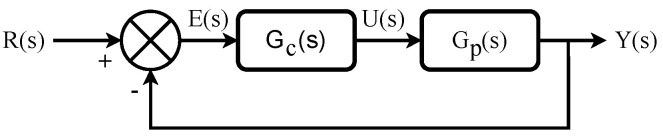
Classical control diagram to regulate voltage in the buck converter of Figure 1.

By turning on and off, the MOSFET *Q* can be operated along with the diode *D* as complementary switches, which allows us to transfer the supply power $V_{i}$ to the load *R* through the inductor *L* when the MOSFET *Q* is on. On the contrary, during the off state, the load *R* receives the energy stored in the inductor *L*. This operation is repeated periodically to achieve at the converter’s output port a regulated voltage which is lower that the one supplied at the input port. The averaged mathematical model describing the above described operation assuming continuous conduction mode and ideal components will be described as follows [36],

(1) $$\begin{array}{c} L\frac{di_{L}}{\mathrm{dt}}=\bar{d}V_{i}-v_{C},\\ C\frac{dv_{C}}{\mathrm{dt}}=i_{L}-\frac{1}{R}v_{C}, \end{array}$$

where $i_{L}$, $v_{C}$ and $\bar{d}$∈(0,1) represent inductor current, capacitor voltage and average of duty cycle *d*, respectively. By considering the classical control diagram in Figure 2 and determining that $v_{C}=v_{o}$, the transfer function from the control law U(s) to the output Y(s), which correspond to the duty cycle *d* and capacitor voltage $v_{C}$, severally, is the converter transfer function that is described as follows,

(2) $$\frac{Y(s)}{U(s)}=G_{p}\left(s\right)=\frac{\left(\frac{V_{i}}{CL}\right)}{s^{2}+\left(\frac{1}{RC}\right)s+\left(\frac{1}{CL}\right)}.$$

**Figure 3:**
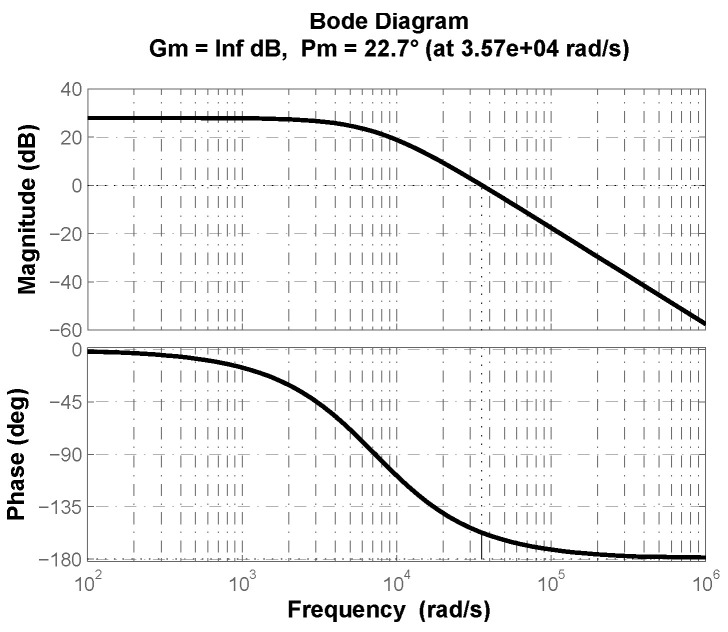
Frequency response of buck converter from Figure 1 and parameter values of Table 1.

By analyzing buck converter transfer function (2) one can determine that the system is of minimum phase since it does not presents right-half plane zeros or poles. The latter can be corroborate through the frequency response of buck converter shown in Figure 3, where no additional phase contribution from zeros or poles can be corroborated and a plant phase $\phi_{p}=-157.3$° in open loop with no control effort is determined.

In the next section, the method to approximate the non-integer order of Laplacian operator through biquadratic modules is described. Some detailed information necessary to fully understand the relevance and advantages of the technique are also provided.

### 2.2. Biquadratic Modules to Fractionally Approximate Laplacian Operator

The approximation proposed in [34,35] employs a quotient of quadratic polynomial to approximate the Laplacian operator $s^{\alpha }$ frequency response, where α∈(0,1), within $\omega_{l}$ and $\omega_{h}$, which represents the approximation validity frequency band.

The transfer function of fractional-order approximation used is described as follows,

(3) $$s^{\alpha }\approx T\left(s\right)=\frac{\left(a_{0}\right)s^{2}+\left(a_{1}\omega_{c}\right)s+\left(a_{2}\omega_{c}^{2}\right)}{\left(a_{2}\right)s^{2}+\left(a_{1}\omega_{c}\right)s+\left(a_{0}\omega_{c}^{2}\right)},$$

which represents a single biquadratic module capable of generating a flattened phase response, where alpha-dependent real constants $a_{0}$, $a_{1}$, $a_{2}$ are given as follows,

(4) $$\begin{array}{ccc} a_{0} & = & \alpha^{\alpha }+3\alpha +2,\\ a_{2} & = & \alpha^{\alpha }-3\alpha +2,\\ a_{1} & = & 6\alpha \;\tan\frac{(2-\alpha )\pi }{4}, \end{array}$$

and $\omega_{c}$ is the frequency around which the approximation’s magnitude and phase curves are centered.

**Figure 4:**
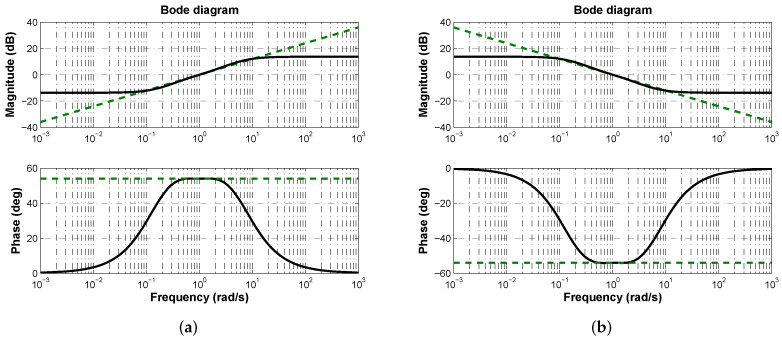
Frequency response of fractional-order approximation (3) for $s^{\pm 0.6}$ where dashed lines represent the theoretical response and solid lines the approximation. (**a**) Derivative effect. (**b**) Integral effect.

By substituting s=jω into (3) the phase contribution of the approximation can be determined as follows,

(5) $$s^{\alpha }\approx T\left(j\omega ,\alpha \right)=\frac{\left(a_{2}-a_{0}\right)+ja_{1}}{-\left(a_{2}-a_{0}\right)+ja_{1}}=\frac{-1+j\tan\frac{(2-\alpha )\pi }{4}}{1+j\tan\frac{(2-\alpha )\pi }{4}}$$

thus, the phase contribution of (3) will be given by,

(6) $$\arg\left\{T\left(j\omega ,\alpha \right)\right\}=-\arctan\left(\tan\frac{(2-\alpha )\pi }{4}\right)-\arctan\left(\tan\frac{(2-\alpha )\pi }{4}\right),$$

which alternates sign as follows,

(7) $$\arg\left\{1/T\left(j\omega ,\alpha \right)\right\}=\arctan\left(\tan\frac{(2-\alpha )\pi }{4}\right)+\arctan\left(\tan\frac{(2-\alpha )\pi }{4}\right).$$

if the inverse of (3) is used. Thus, one can conclude that the phase contribution of fractional-order approximation will be given by,

(8) $$\arg\left\{s^{\pm \alpha }\right\}=\pm \alpha \frac{\pi }{2},$$

which depends on the value of α. Therefore, the phase contribution of a single biquadratic module can be modulated from −90° to 90° depending on the desired effect, which can be derivative or integral. Figure 4 shows the frequency response of approximation (3) for both derivative and integral effects when α=0.6, which correspond to a phase contribution of ±54°.

**Figure 5:**
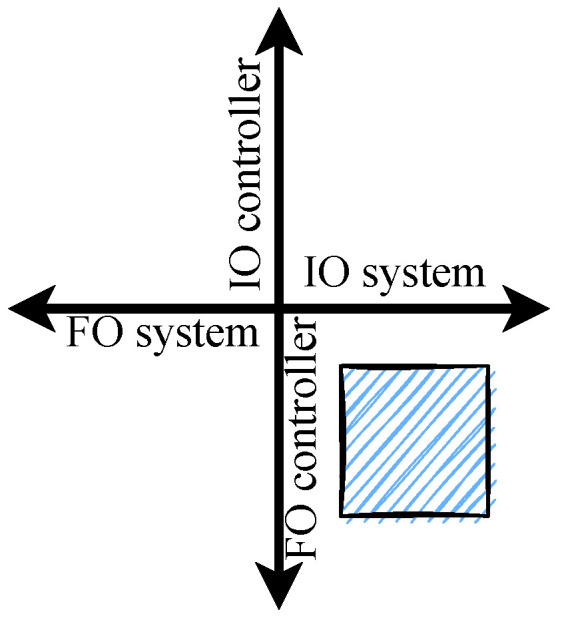
Possible combinations for integer-order (IO) and fractional-order (FO) approaches for the system-controller duo when introducing Fractional Calculus into Control Theory.

Note that the Laplacian operator approximation will be performed over the controller structure as will be shown and explained in the following section. Thus, in this manuscript integer and fractional approaches are combined as shown in Figure 5, which is one of the four possible scenarios that can be explored when introducing Fractional Calculus into Control Theory.

In the following section, two nature-inspired optimization algorithms are briefly reviewed. Basics on these methods such as physical interpretation, parameters and operation conditions are described. Lastly, importance of minimization criterion and variables restrictions are also mentioned.

### 2.3. Definition of Minimization Criteria: An Error-Based Approach

From an engineering point of view, a very basic and general idea of optimization can be given as the process of finding the conditions for a system to operate as efficiently and smoothly as possible. Such conditions are known as the best solution to the problem and imply the evaluation of at least a minimization/maximization criterion and a set of constraints related.

Bio-inspired optimization algorithms are some of the most used techniques to effectively perform this searching in engineering-related problems. From the computational intelligence field, swarm-based and evolutionary algorithms are the preferred ones to achieve optimal solutions [37]. Particle swarm and genetic algorithms are the most accepted and widely used as optimization methods in engineering problems. Physical interpretation, easy coding, preserving search information over iterations, not gradient data required, fast convergence and bypassing local optima are some of their most notable characteristics [37,38].

Finding the appropriate balance between the optimization algorithm’s main capabilities/operators, i.e., exploration and exploitation for the particle swarm optimization algorithm or crossover and mutation from genetic one, along with the definition of a suitable minimization criterion and the pertinent set of constrains represent critical aspects to guarantee fast convergence and global minimum. The most used parameter to define minimization criteria in control problems is the error e(t), which has been minimized by integrating either the error itself, its square value, its absolute value or its square absolute value [19].

For this case, the following two error-based criteria will be used to tune controller parameters,

(9) $$J_{1}:=\int_{t_{0}}^{t}\left|e\left(\tau \right)\right|d\tau ,$$

and

(10) $$J_{2}:=\lim_{s\to 0}\frac{1}{1+G_{o}\left(s,\alpha \right)},$$

both restricted to

(11) $$\begin{array}{ccc} \phi_{d} & = & \pi /3,\\ \alpha & = & (-\pi +\phi_{d}+\phi_{p})/\pi /2, \end{array}$$

where $G_{o}\left(s,\;\alpha \right)$ is the open-loop transfer function of control diagram from Figure 2 and $\phi_{d}$ is the system’s desired closed-loop phase margin. Note that $J_{2}$ is the simplified form of closed-loop steady-state error expression for the input R(s) a step. Criteria $J_{1}$ and $J_{2}$ quantify the error from different perspectives and allow us to determine best possible controller parameters based on which provides the smaller value.

Note that constraints (11) seek to ensure robustness of closed-loop system without compromising viability of controller’s implementation. First constraint is intended to guarantee robustness by setting the desired phase margin to the upper limit of the acceptable range commonly considered within [π/6, π/3] [39]. Second restriction prevents optimization algorithms from consider α values that produce high-gain controllers, which in turn would require non-commercial/high-value/expensive components that derive in saturated control laws.

Global minimum for the optimization problem is guaranteed as long as $J_{1}$, $J_{2}$ and constraint (11) are linear [40] (Chaps. 10, 11). $J_{1}$ is defined in terms of an integral, which is a linear operator. The error is given by e(τ)=r(τ)−y(τ), whose solution curve can be determined through its *s*-domain representation $E\left(s\right)=\frac{1}{1+G_{o}\left(s,\;\alpha \right)}R\left(s\right)$. $J_{2}$ is based on the steady-state error, thus it is linear also. Linearity of constraints is determined directly by analyzing their structure.

Particle swarm and genetic optimization algorithms will be used to minimize criteria (9) and (10) with constraints (11). A comparison from the obtained results will be performed to determine those that produce the most effective plant response.

In the following section, the fractional approximation of controller structure as well as numerical and implementation results are provided and described.

## 3. Results

In this section some mathematical considerations and derivations to determine controller structure are described. Numerical simulations and results of experimental validation are provided to corroborate the proposed controller effectiveness.

### 3.1. Two-Modes Controller Structure

The general expression of a two-modes controller structure is either a Proportional-Integral (PI) described by $G_{c}\left(s\right)=k_{p}\left(1+\frac{1}{T_{i}s}\right)$ or a Proportional-Derivative (PD) given by $G_{c}\left(s\right)=k_{p}\left(1+T_{d}s\right)$, that can be modified to integrate the fractional-order approach as follows,

(12) $$G_{c}\left(s,\;\alpha \right)=k_{p}\left(1+T_{d}s^{\alpha }\right),$$

for the PD controller and

(13) $$G_{c}\left(s,\;\alpha \right)=k_{p}\left(1+\frac{1}{T_{i}s^{\alpha }}\right),$$

for the PI structure, from which the fractional-order Laplacian operator $s^{\alpha }$ can be identified and $k_{p}$, $T_{d}$, $T_{i}$ are proportional gain, derivative and integral time constants, respectively.

To determine if a PD or PI controller is required for the plant under consideration, it is necessary to analyze frequency information previously provided as follows,

1. The uncontrolled plant phase is $\phi_{p}=-157.33$° (Figure 3). From $\phi_{p}=-\pi +\phi_{m}$ it is deduced that phase margin is $\phi_{m}=22.67$° (Figure 3).
2. If desired phase margin is $\phi_{d}=\pi /3=60^{{}^{\circ}}$, from $\phi_{c}+\phi_{p}=-\pi +\phi_{d}$ it is deduced that controller phase contribution has to be $\phi_{c}=37.33$°.
3. Due to $\phi_{c}>0$, the fractional-order Laplacian operator has to be approximated to behave as shown in Figure 4a. Therefore, the controller structure must be a PD as in (12).

By substituting (3) into (12), the approximation of two-modes controller structure will be given as follows,

(14) $$G_{c}\left(s,\;\alpha \right)=\frac{k_{p}\left(D\left(s,\;\alpha \right)+T_{d}N\left(s,\;\alpha \right)\right)}{D(s,\;\alpha )},$$

where N(s,α) and D(s,α) are numerator and denominator of fractional-order approximation of Laplacian operator (3).

The parameters of PD controller (14) will be tuned through swarm and genetic optimization algorithms by minimizing criteria (9) and (10) with constraints (11). It is worth noting up to this point that once both controller and plant transfer functions are know, criterion $J_{2}$ can be simplified by computing open-loop transfer function $G_{o}\left(s,\;\alpha \right)$ of control diagram from Figure 2 as follows,

(15) $$G_{o}\left(s,\;\alpha \right)=\frac{\left(\frac{V_{i}}{CL}\right)N_{c}\left(s,\;\alpha \right)}{\left(s^{2}+\left(\frac{1}{RC}\right)s+\left(\frac{1}{CL}\right)\right)D_{c}\left(s,\;\alpha \right)},$$

where $N_{c}\left(s,\;\alpha \right)$ and $D_{c}\left(s,\;\alpha \right)$ are numerator and denominator of PD controller (14), severally. If the error is defined as E(s,α)=R(s)−Y(s,α) and the closed-loop transfer function is given by $\frac{Y(s,\;\alpha )}{R(s)}=G\left(s,\;\alpha \right)=G_{o}\left(s,\;\alpha \right)/\left(1+G_{o}\left(s,\;\alpha \right)\right)$, the mathematical model of closed-loop error will be described as follows,

(16) $$E\left(s,\;\alpha \right)=\frac{1}{1+G_{o}\left(s,\;\alpha \right)}R\left(s\right),$$

thus, the closed-loop steady-state error can be computed as,

(17) $$e_{ss}=\lim_{s\to 0}sE\left(s,\;\alpha \right),$$

which can be simplified to

(18) $$J_{2}\equiv e_{ss}=\frac{\alpha^{\alpha }+3\alpha +2}{\left(b_{1}+b_{2}\right)\alpha^{\alpha }+3\left(b_{1}-b_{2}\right)\alpha +2\left(b_{1}+b_{2}\right)},$$

where $b_{1}=\left(1+k_{p}V_{i}\right)$ and $b_{2}=k_{p}T_{d}V_{i}$.

In the following section, numerical results from optimization and voltage regulation of buck converter are described. Experimental validation to corroborate viability of proposed approach is provided as well.

### 3.2. Numerical Results

**Figure 6:**
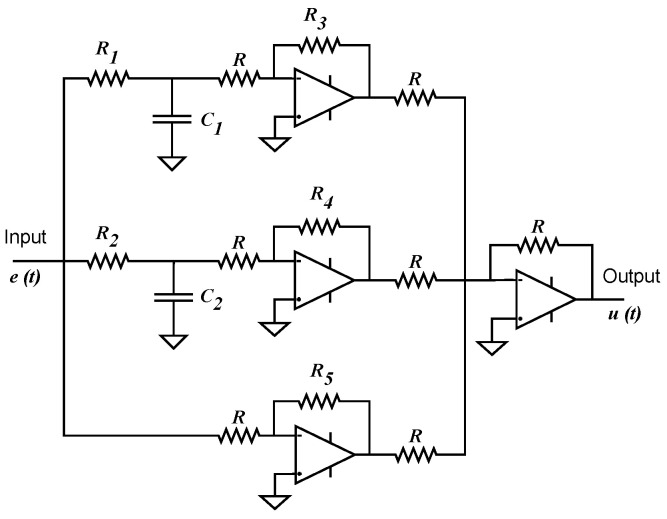
Representation of electrical circuit to implement partial fraction expansion (20) of PD controller (19).

Once optimization algorithms have been applied to minimize criteria (9) and (10) with constraints (11), the PD controller (14) will be given by the following transfer function,

(19) $$G_{c}\left(s\right)=k_{c}\frac{s^{2}+\beta_{1}s+\beta_{2}}{s^{2}+\beta_{3}s+\beta_{4}},$$

with its partial fraction expansion given by,

(20) $$G_{c}\left(s\right)=\left(\frac{R_{3}/R}{R_{1}C_{1}s+1}\right)+\left(\frac{R_{4}/R}{R_{2}C_{2}s+1}\right)+\frac{R_{5}}{R},$$

due to the roots of denominator polynomial will always be real because $a_{1}^{2}>4a_{2}a_{0}$ holds ∀α∈(0,1). Implementation of (20) would require two RC circuits and operational amplifiers connected as shown in Figure 6.

Operation conditions for both algorithms were set as follows:

1. Particle swarm optimization algorithm (PS)
  - Iterations: 600
  - Population: 90
  - Inertia coefficient *w*: 1
  - Cognition constant $c_{1}$: 2
  - Social constant $c_{2}$: 2
2. Genetic optimization algorithm (GA)
  - Iterations: 600
  - Population: 90
  - Mutation rate: 0.25
  - Random recombination.
  - Scale population coefficient: 1

Numerical results obtained from optimization process are shown in Table 2 for both minimization criteria (9) and (10) with constraints (11). Data summarized in Table 2 were obtained from 150 runs performed with each optimization algorithm for each minimization criterion $J_{1}$ and $J_{2}$.

Form Table 2 we can observe more than one solution when minimizing $J_{1}$, however one can note that every solution converge essentially to the same minimum for both optimization algorithms, since $k_{p}$ and $T_{d}$ vary only in the order of thousandths. On the other hand, when minimizing $J_{2}$ a non-negligible difference between possible solutions obtained from both optimization algorithms is determined. Note that minima are essentially the same for either algorithm but different between them. As will be shown later on, this small difference results in smaller control laws.

By substituting $\left[\alpha ,\;k_{p},\;T_{d}\right]$ from Table 2 into the controller structure (14), parameters of transfer function (19) and component values for its partial fraction expansion (20) are provided in Table 3 and Table 4, respectively.

**Figure 7:**
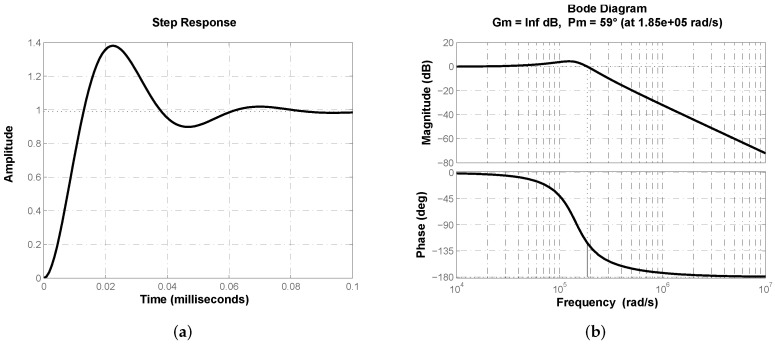
(**a**) Closed-loop step response of plant’s transfer function (2) with fractional-order approximation (19). (**b**) Frequency response corroborating closed-loop phase margin $\phi_{d}\approx 60^{{}^{\circ}}$.

From Table 3 one can see that optimization results for both algorithms when minimizing criterion $J_{1}$ are essentially the same with small variations in the controller’s gain $k_{c}$. In Figure 7a the closed-loop step response of buck converter transfer function (2) with controller (19) is shown. Effectiveness of proposed structure regulating output voltage in buck converter can be corroborated. Response velocity can be characterized by its time-related performance parameters rise time $t_{r}=8.72$ μs, peak time $t_{p}=22.33$ μs and settling time $t_{s}=76.66$ μs. Figure 7b depicts closed-loop system’s frequency response where desired phase margin $\phi_{d}\approx 60$° can be corroborated.

**Figure 8:**
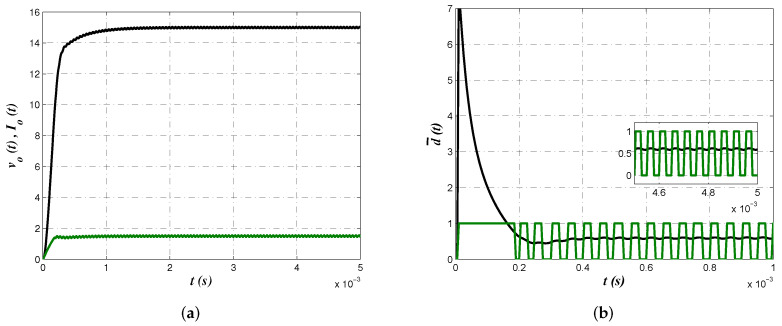
(**a**) Buck converter output voltage $v_{o}\left(t\right)$ (black) and inductor current $i_{L}\left(t\right)$ (green) to corroborate regulation and continuous conduction mode operation. (**b**) Control law $\bar{d}\left(t\right)=0.6$ and its effect on the PWM signal.

Numerical simulations from PSIM 9.0 software allow us to corroborate proposed approach effectiveness from the electrical perspective. In Figure 8 buck converter output voltage $v_{o}\left(t\right)$, inductor current $i_{L}\left(t\right)$ and control law $\bar{d}\left(t\right)$ are shown. In Figure 8a voltage regulation with a smooth convergence to the reference value as well as continuous conduction mode can be confirmed. On the other side, Figure 8b depicts the control law where its convergence to $\bar{d}=0.6$ and the corresponding effect on the pulse width modulation (PWM) signal can be corroborated.

**Figure 9:**
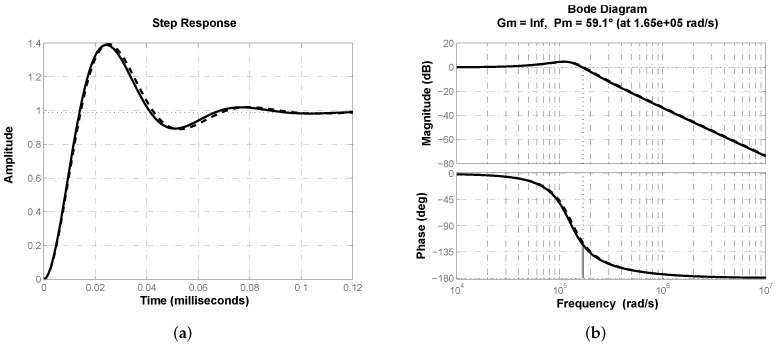
(**a**) Closed -loop step response of plant’s transfer function (2) with fractional-order approximation (19). (**b**) Frequency response corroborating closed-loop phase margin $\phi_{d}\approx 60^{{}^{\circ}}$.

On the other side, when optimizing through the minimization of criterion $J_{2}$, values of resistances $R_{3}$, $R_{4}$ and $R_{5}$ vary from the previous case, thus it is necessary to validate they are appropriate. In Figure 9a the output of buck converter regulated with fractional-order PD controller approximation (19) is shown. System’s frequency response is shown in Figure 9b to corroborate desired phase margin $\phi_{d}\approx 60$°. Note that despite small variations of the values, output behavior and frequency responses are very similar.

**Figure 10:**
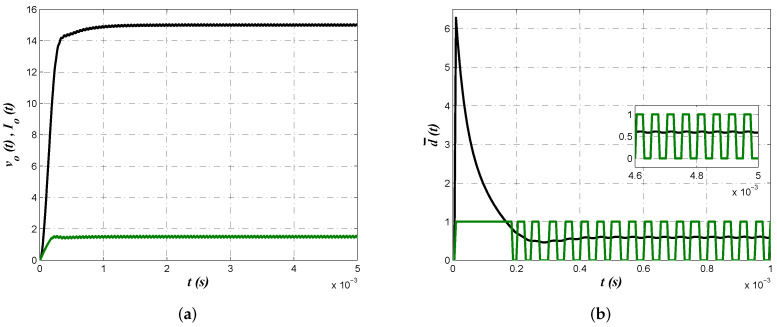
(**a**) Buck output voltage $v_{o}\left(t\right)$ (black) and inductor current $i_{L}\left(t\right)$ (green) to determine effective regulation and converter continuous conduction mode operation. (**b**) Control law $\bar{d}\left(t\right)=0.6$ and its effect on the PWM signal.

Electrical simulations allow us to determine effectiveness of the results obtained from second minimization criterion $J_{2}$. Figure 10 shows converter output voltage $v_{o}\left(t\right)$, inductor current $i_{L}\left(t\right)$ and control law $\bar{d}\left(t\right)$ as in the previous case. Smooth convergence of converter output voltage and inductor current that corroborates continuous conduction mode are shown in Figure 10a. Evolution of control law to its average value and the corresponding effect on the PWM signal are shown in Figure 10b.

**Figure 11:**
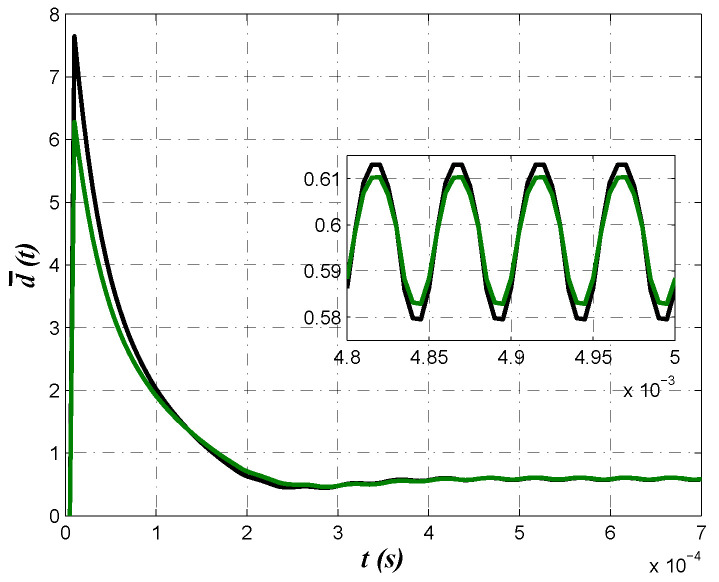
Control laws comparison for both optimization algorithms when minimizing criteria $J_{1}$ and $J_{2}$.

A comparison between control laws obtained from minimization of criteria $J_{1}$ and $J_{2}$ are shown in Figure 11. Note that despite behavior of output voltage shown in Figure 8a and Figure 10a are very similar, control law produced by minimization of the second criterion $J_{2}$ was smaller. This is attributed to the values of resistance $R_{3}$ and $R_{5}$, which are smaller for both optimization algorithm when minimizing $J_{2}$, which is the reason to choose these component values to be implemented.

In order to make evident the relevance, importance and impact of considering controller implementation viability in the optimization process, minimization of criteria $J_{1}$ and $J_{2}$ was performed without considering second constraint over α from (11). In Table 5 optimized parameters, controller coefficients and component value for (12), (19) and (20) are summarized, severally.

As in the previous results, similarity between obtained parameter values is determined when comparing optimization algorithms for a particular minimization criterion. On the other hand, significant difference can be observed when comparing results between minimization criteria, particularly in derivative time constant $T_{d}$. Another singularity of these results is the obtained value for α, which resulted more than double the previous one. Recalling from Section 2.2 that controller contribution can be within ±90°, α≈1 would imply that almost all the phase contribution of the biquadratic module is required, which would be imprecise if an uncontrolled plant such as (2) with $\phi_{p}=-157.3$° is under consideration.

Note that optimized parameter values for α, $k_{p}$ and $T_{d}$ resulted in a considerable increase for controller coefficients of structure (19). It is worth noting the obtained values for controller gain $k_{c}$, which confirms that optimizing without restriction over the approximation order undoubtedly leads to the synthesis of a high-gain controller as previously stated. Remarkable differences can be observed in component values for partial fraction expansion (20) and its corresponding implementation circuit from Figure 6, where gains to generate first and third terms are considerably big, resulting in resistance values of MΩ.

Lastly, a comparison of the proposed approach with its integer-order counterpart allows us to determine that the fractional-order controller approximation represents an alternative to achieve voltage regulation in a buck converter. By using minimization criteria $J_{1}$, $J_{2}$ and only the constraint on phase margin $\phi_{d}$ from (11), parameters of a classical PD controller were optimized with both algorithms. Optimization results are summarized in Table 6 for $k_{p}$, $T_{d}$ and the corresponding parameter values required to implement the PD controller, where $R_{i}$/$R_{f}$ are input and feedback resistances of the operational amplifier generating the proportional model and $C_{i}$/$R_{f}$ are input capacitor and feedback resistance for operational amplifier generating the derivative mode.

**Figure 12:**
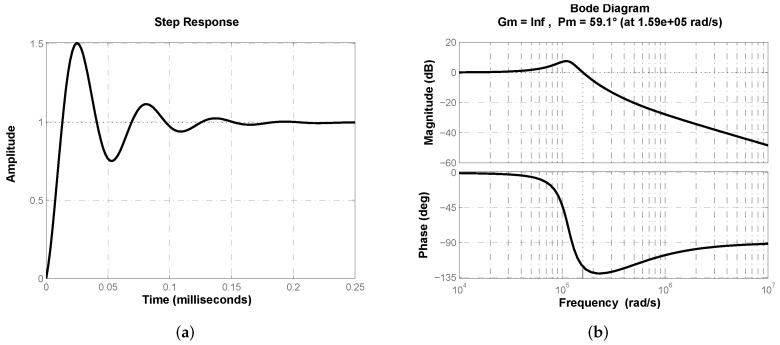
(**a**) Closed-loop step response of plant’s transfer function (2) with classical PD controller $G_{c}\left(s\right)=kp\left(1+T_{d}s\right)$. (**b**) Frequency response corroborating closed-loop phase margin $\phi_{d}\approx 60^{{}^{\circ}}$.

In Figure 12a the closed-loop step response of buck converter transfer function (2) with a classical PD controller $G_{c}\left(s\right)=kp\left(1+T_{d}s\right)$ is shown. Figure 12b depicts closed-loop system’s frequency response where desired phase margin $\phi_{d}\approx 60^{{}^{\circ}}$ can be corroborated. Thus a comparison through performance parameters of both responses from Figure 7a and Figure 12a is valid and allows us to determine advantages of proposed approach. Table 7 summarizes step response performance parameters for both control schemes, from which superiority of fractional-order PD controller approximation can be determined.

In the following section, experimental validation is provided as evidence of proposed approach effectiveness. As will be seen, behavior of output voltage $v_{o}\left(t\right)$ and control law $\bar{d}\left(t\right)$ from Figure 8 and Figure 10 will be corroborated.

### 3.3. Experimental Results

In this section, experimental results from the implementation of closed-loop control diagram shown in Figure 2 will be provided, where the plant to be controlled is the buck converter of Figure 1, whose input-to-output relation is given by (2), and transfer function of controller fractional approximation given by (19), whose electrical circuit is depicted in Figure 6.

**Figure 13:**
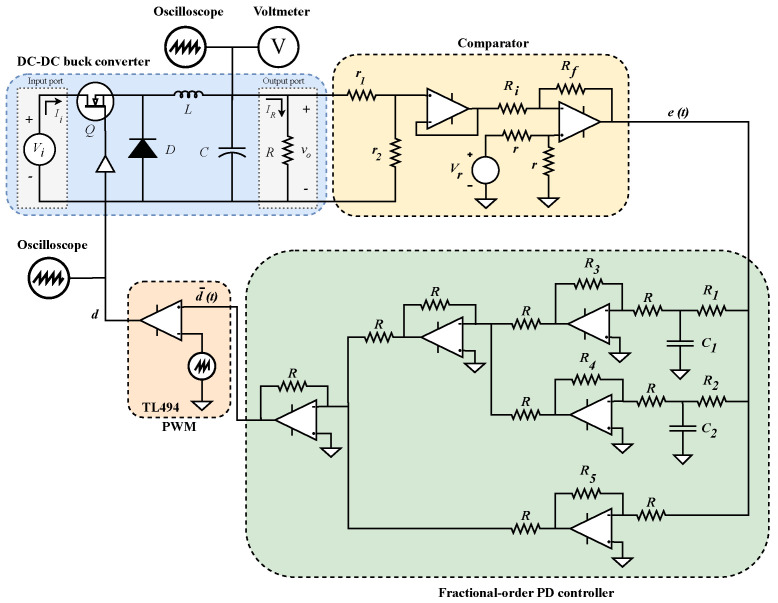
Electrical arrangement for implementation of control diagram from Figure 2 where plant, controller, comparator and PWM blocks can be identified.

The electrical arrangement representing the physical implementation of the control diagram is depicted in Figure 13. The plant to be controlled is the buck converter, implemented as previously described, shown in the blue square. Fractional-order approximation of PD controller is shown in the green square with its corresponding interconnections. The diagram’s comparison block is shown in the yellow square. Comparator was implemented through a voltage divider, where $r_{1}=24$ kΩ, $r_{2}=1$ kΩ to produce a gain of $k_{d}=1/25$, and an operational amplifier in difference configuration with resistance values $R_{i}=R_{f}=r=1$ kΩ to generate $e=V_{r}-k_{d}v_{o}$ signal, where $V_{r}=0.6$ V. A pulse-width-modulator control circuit TL494 was used for the PWM signal and a 4 MHz operational amplifiers LF347N for comparator and controller.

Voltage measurements were made with a four-channel Tektronix TDS 2024C oscilloscope. Current measurements were made with a Tektronix—A622 AC/DC 100 mV/A current probe.

**Figure 14:**
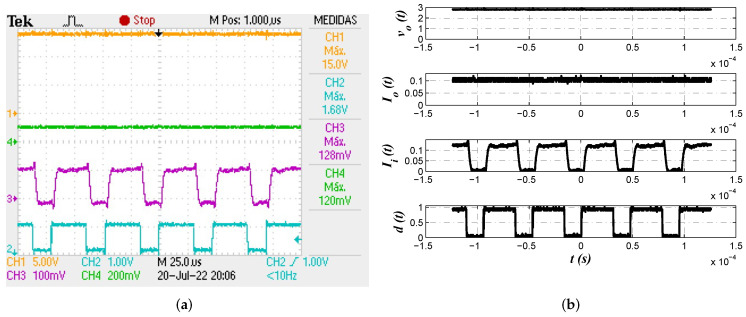
(**a**) Oscilloscope view for measurements of output voltage $v_{o}\left(t\right)$ (yellow), output current $I_{o}\left(t\right)$ (green), input current $I_{i}\left(t\right)$ (purple) and PWM signal d(t) (cyan). (**b**) Alternative view of exported experimental data preserving scale of 5 V/unit for $v_{o}\left(t\right)$, 100 mV/unit for $I_{o}\left(t\right)$ and $I_{i}\left(t\right)$.

Voltage regulation was the first test performed over the circuit of experiment from Figure 13. As previously stated, reference voltage was set to $V_{r}=0.6$ V, which is expected to produce a voltage of $v_{o}\left(t\right)=15$ V in the converter. In Figure 14a output voltage $v_{o}\left(t\right)$, output current $I_{o}\left(t\right)$, input current $I_{i}\left(t\right)$ and PWM signal d(t) are shown. As one can see, the controller fractional approximation (19) effectively achieved buck converter output to reach the specified value. In Figure 14b an alternative view of measurements made from data exported is shown. Scales for $v_{o}\left(t\right)$, $I_{o}\left(t\right)$, $I_{i}\left(t\right)$ and d(t) were preserved for comparative purposes.

**Figure 15:**
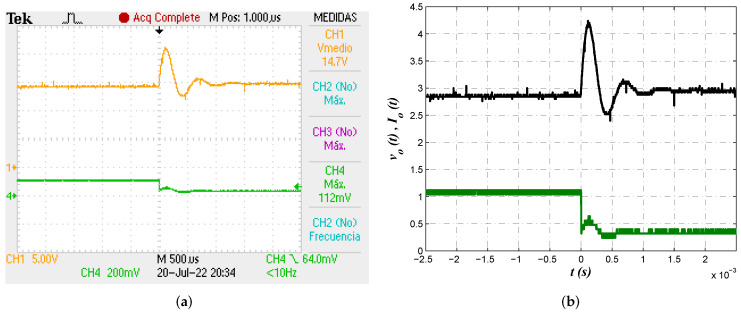
(**a**) Oscilloscope view for measurement of output voltage $v_{o}\left(t\right)$ and load current $I_{o}\left(t\right)$ in the presence of load variation. (**b**) Alternative view of output voltage $v_{o}\left(t\right)$ and load current $I_{o}\left(t\right)$ from exported data.

Second test performed over the implemented circuit was output regulation in the presence of load variation, for which the value of resistance was changed from $R_{o}=10$ Ω to $R_{1}=45$ Ω. The objective is to determine controller approximation’s effectiveness of keeping the voltage level at the reference value. Figure 15 shows the evolution of output voltage $v_{o}\left(t\right)$ and load current $I_{o}\left(t\right)$ in the presence of load variation. Efficiency of proposed approach to return the output voltage to reference level was corroborated, since it took the controller about 1 ms to restore the voltage level.

**Figure 16:**
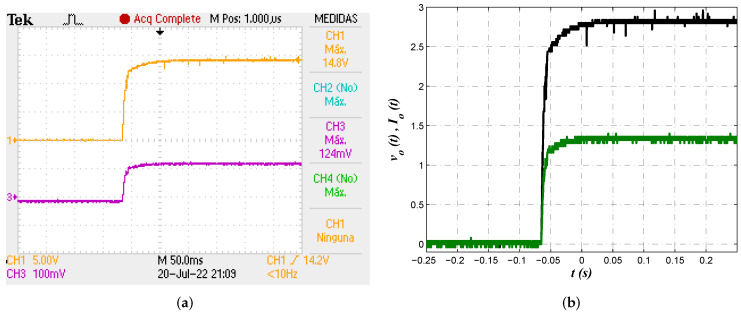
(**a**) Oscilloscope view for measurement of reference tracking characteristic from output voltage $v_{o}\left(t\right)$ and the corresponding evolution of load current $I_{o}\left(t\right)$. (**b**) Alternative view of output voltage $v_{o}\left(t\right)$ and load current $I_{o}\left(t\right)$ from exported data, preserving scale of 5 v/unit for $v_{o}\left(t\right)$.

Lastly, the reference tracking characteristic of the system was tested to corroborate behavior described by numerical data provided in Figure 8a and Figure 10a. As was predicted by the results of electrical simulations, it is expected the output voltage $v_{o}\left(t\right)$ to evolve smoothly. In Figure 16 the reference tracking characteristic of output voltage $v_{o}\left(t\right)$ can be confirmed through experimental measurements. Stable regulation and smooth convergence to the reference value can be observed.

Relevant results from the section can be summarized as follows: firstly, the proposal of $J_{2}$ as minimization criterion, which focuses on the difference between reference and output in steady state rather than its accumulated value. Note that it is entirely defined as function of controller and plant parameters which makes it easier to compute. Tightly related are the proposed constraints, which seek to guarantee closed-loop system’s robustness through $\phi_{d}$, but limiting it in such a way that viability of controller’s implementation is not compromised, thus preventing from synthesizing high-gain controllers that produce saturated control laws. Note that constraint imposed over α is intended to ensure that controller phase contribution is limited only to what it is necessary to achieve $\phi_{d}$. As was demonstrated through numerical simulation and experimental validation, the combination of both constraints derived in the synthesis of an implementable fractional-order PD controller approximation, which effectively regulated output voltage $v_{o}\left(t\right)$ of a buck converter.

Secondly, from Table 2 one can conclude that combination of two optimization algorithms with two different minimization criteria allows us to corroborate that both methods converge to a neighborhood of the point in search space that produces the minimum value for the criteria, since $k_{p}$ and $T_{d}$ are very similar between algorithms. On the other hand, a small differences can be observed in $k_{p}$ and $T_{d}$ when comparing between $J_{1}$ and $J_{2}$.

Thirdly, from Table 3 coefficient values of controller transfer function (19) for denominator are equal regardless the optimization algorithm or minimization criteria. Numerator coefficients of (19) vary slightly between $J_{1}$ and $J_{2}$ but are very similar when comparing optimization algorithms. Note that biggest difference is in controller’s gain $k_{c}$, being proposed criterion $J_{2}$ the one that leads to the smallest value.

These similarities resulted in controller component values of Table 4, from which can be seen that RC networks can be generated with the same resistance values $R_{1}$ and $R_{2}$ regardless optimization algorithm or minimization criterion. On the other side, resistance values $R_{3}$, $R_{4}$ and $R_{5}$ are very similar between algorithms and with small variations when comparing minimization criteria. By analyzing controller structure of Figure 6 and component values from Table 4 one can determine that required derivative effect is generated by $R_{3}$ and $R_{4}$ while proportional effect by $R_{5}$, whose value is directly related with controller’s gain $k_{c}$.

In the following sections some discussion on the presented results and conclusions are provided.

## 4. Discussion

In the present work, viability of a two-modes controller fractional-order approximation to regulate output voltage of a buck converter was investigated. Bio-inspired optimization algorithms along with two error-based minimization criteria (9) and (10) were used to determine controller parameters. Optimization constraints (11) were intended to incorporate controller’s implementation viability, thus avoiding synthesis of high-gain ones that produce saturated control laws.

Proposal of minimization criterion $J_{2}$ represents an alternative that has not been explored since, as early mentioned, integral of error itself, its absolute/square absolute or different versions of its weighted absolute values are the preferred ones. For the purpose of this study, proposed minimization criterion resulted in the synthesis of controller structures with lower gains, which in turn generated smaller control laws.

The idea of incorporating constraints that impact implementation represents a novelty due to it is common to let the algorithm to determine from the search space those values that minimize criteria without considering the impact on controller implementation. This fact takes special relevance when controlling power converters. Due to the PWM signal d(t) is generated by comparing controller’s output $\bar{d}\left(t\right)$ and a sawtooth signal whose value oscillates between 0.5–3.5 V, it is inconvenient and not very useful generating a control effort of tens of volts, which commonly characterizes a high-gain controller’s control law, to achieve the modulation that can be done with a smaller signal. In addition, bigger control laws $\bar{d}\left(t\right)$ increase time the MOSFET is in the ON state during the transient response, which represents a serious problem when implementing converters such as boost or buck-boost due to inductor is directly connected with the power supply during the ON state. Thus, generating and using the smallest control law $\bar{d}\left(t\right)$ possible considerably improves implementation stage.

## 5. Conclusions

A PD controller fractional-order approximation was synthesized and tuned through bio-inspired optimization algorithms to achieve output regulation in a buck converter. The optimization procedure included the proposal of an alternative error-based minimization criterion $J_{2}$ in combination with two constraints that were intended to ensure system’s robustness while preserving controller’s implementation viability.

Particle swarm and genetic optimization algorithms were used to determined controller parameters. The integral of absolute value and the steady state value of the error were criteria to be minimized. Constraint over phase margin $\phi_{d}$ sought to ensure robustness of closed-loop system. Second constraint avoids compromising controller’s implementation viability by limiting tuning parameters to those values that produce only the controller phase contribution needed to achieve the required $\phi_{d}$.

Optimization results showed that both algorithms converge to similar parameter values for a specific minimization criterion. Small differences were observed when comparing optimization results between both minimization criteria (Table 2). This behavior was repetitive in controller coefficients computation (Table 3) and implementation component values (Table 4). It is remarkable from Table 4 that smaller component values were obtained when optimizing with proposed minimization criteria, thus resulting in smaller control laws.

A comparison between the proposed approach and its integer-order counterpart allowed us to determine viability, effectiveness and superiority of the fractional-order PD controller approximation. By using performance parameters of both step responses, faster regulation velocity from the proposed approach was confirmed. In addition, the overshoot was smaller in when the fractional-order PD controller was used.

Experimental data confirmed numerical simulations, where proposed approach effectiveness to regulate buck converter output voltage $v_{o}\left(t\right)$ was predicted. In addition, reference tracking and regulation in the presence of load variation were determined.

Future direction of this work seem to be the proposal of a control strategy that considers not only robustness and performance but also disturbance rejection in a multi-objective optimization scheme, a multi-loop or current control mode approach to take advantage of convergence velocity from second variable.

## Figures and Tables

**Table 1:** Parameter values for the implementation of buck converter in Figure 1.

Component/Element	Notation	Value	Generals
Capacitor	*C*	7 μF	C4AQCBU4700A1YJ, 650 V, ±5%
Inductor	*L*	2.7 mH	1140-272K-RC, 555 mΩ, 2.2/3.9 A, ±10%
Resistance	*R*	10 Ω	CB25JB10R0, 25 W, ±5%
Power supply	$V_{i}$	25 V	Programmable BK Precision 9129B
MOSFET	*Q*		PSMN022-30PL, N-Ch, 30 V , 22 mΩ, 30 A
Diode	*D*		MUR840, 50–600 V, 8 A, 1 V
MOSFET driver			Optocoupler TLP250
Switching frequency	$f_{sw}$	20 kHz	

**Table 2:** Optimization results from applying particle swarm and genetic algorithms to minimization criteria (9) and (10) with constraints (11).

	$J_{1}$			$J_{2}$		
	α	$k_{p}$	$T_{d}$	α	$k_{p}$	$T_{d}$
GA	0.4148	1.2839	4.9995	0.4148	1.9331	2.5497
	0.4148	1.2847	4.9995	0.4148	1.9225	2.5553
	0.4148	1.2842	4.9995	0.4148	1.7595	3.0316
	0.4148	1.2851	4.9994			
	0.4148	1.2856	4.9927			
	0.4148	1.2844	4.9989			
PS	0.4148	1.2851	5	0.4148	1.8759	2.7445
	0.4148	1.2852	5	0.4148	1.8773	2.7404
	0.4148	1.2854	5	0.4148	1.8733	2.7522

**Table 3:** Computed values for coefficients of controller’s transfer function (19).

	$J_{1}$			$J_{2}$		
	$\beta_{1}$^1^/$\beta_{2}$^2^	$\beta_{3}$^3^/$\beta_{4}$^4^	$k_{c}$	$\beta_{1}$^1^/$\beta_{2}$ ^2^	$\beta_{3}$^3^/$\beta_{4}$ ^4^	$k_{c}$
GA	7.461/6.739	1.813/3.46	18.7218	8.121/8.461	1.813/3.46	15.3230
	7.461/6.739	1.813/3.46	18.7334	8.118/8.454	1.813/3.46	15.2683
	7.461/6.739	1.813/3.46	18.7261	7.916/7.926	1.813/3.46	16.2504
	7.461/6.739	1.813/3.46	18.7389			
	7.462/6.741	1.813/3.46	18.7228			
	7.461/6.739	1.813/3.46	18.7270			
PS	7.461/6.739	1.813/3.46	18.7410	8.03/8.225	1.813/3.46	15.8624
	7.461/6.739	1.813/3.46	18.7425	8.032/8.229	1.813/3.46	15.8533
	7.461/6.739	1.813/3.46	18.7454	8.027/8.216	1.813/3.46	15.8796

^1^ ×10^4^. ^2^ ×10^8^. ^3^ ×10^5^. ^4^ ×10^9^.

**Table 4:** Computed values for components of controller’s partial fraction expansion (20) and corresponding electrical circuit of Figure 6, where $C_{1}=C_{2}=2.2$ μF and R=1 kΩ. Units for $R_{1}$ and $R_{2}$ are given in Ω. Units for $R_{3}$, $R_{4}$ and $R_{5}$ are given in kΩ.

	$J_{1}$		$J_{2}$	
	$R_{1}$/$R_{2}$	$R_{3}/R_{4}/R_{5}$	$R_{1}$/$R_{2}$	$R_{3}/R_{4}/R_{5}$
GA	13.78/101.54	12.11/2.96/18.72	13.78/101.54	9.30/2.28/15.32
	13.78/101.54	12.12/2.97/18.73	13.78/101.54	9.27/2.27/15.27
	13.78/101.54	12.12/2.96/18.73	13.78/101.54	10.07/2.46/16.25
	13.78/101.54	12.12/2.97/18.74		
	13.78/101.54	12.11/2.96/18.72		
	13.78/101.54	12.12/2.96/18.73		
PS	13.78/101.54	12.13/2.97/18.74	13.78/101.54	9.72/2.38/15.86
	13.78/101.54	12.13/2.97/18.74	13.78/101.54	9.71/2.38/15.85
	13.78/101.54	12.13/2.97/18.75	13.78/101.54	9.7/2.38/15.87

**Table 5:** Optimized parameters, controller coefficients and component values from minimizing criteria (9) and (10) without constraint over α. Units for $R_{1}$, $R_{2}$ are in Ω, $R_{3}$, $R_{5}$ in MΩ and $R_{4}$ in kΩ.

	$J_{1}$			$J_{2}$		
	Optimization results					
	α	$k_{p}$	$T_{d}$	α	$k_{p}$	$T_{d}$
GA	0.9936	29.728	29.8142	0.975	29.9437	7.8793
	0.9936	29.9927	29.9945	0.9396	29.9912	3.2028
	0.9937	29.9999	29.9975	0.846	29.9346	1.2147
	0.9937	29.9931	29.9971	0.8917	29.9948	1.7824
	0.9936	30	29.9904	0.9762	29.9868	8.4766
	0.9936	29.9959	29.9357	0.9489	29.978	3.9742
PS	0.9937	30	30	0.8847	30	1.7319
				0.8843	30	1.7255
				0.8786	30	1.607
				0.8889	30	1.8075
	Controller coefficients					
	$\beta_{1}$^1^/$\beta_{2}$ ^2^	$\beta_{3}$^2^/$\beta_{4}$ ^3^	$k_{c}$ ^1^	$\beta_{1}$^1^/$\beta_{2}$^2^	$\beta_{3}$^4^/$\beta_{4}$ ^5^	$k_{c}$ ^6^
GA	3.717/4.545	1.674/5.925	41.241	4.142/17.24	42.9/14.85	27.534
	3.717/4.519	1.674/5.925	41.859	5.005/42.22	17.79/5.901	4.4812
	3.716/4.514	1.7/6.02	42.543	7.426/107.2	7.007/2.086	0.6255
	3.716/4.514	1.7/6.02	42.533	6.197/74.93	9.946/3.119	1.3394
	3.717/4.52	1.674/5.925	41.864	4.104/16.05	45.05/15.62	31.198
	3.717/4.527	1.674/5.925	41.782	4.731/34.19	21.02/7.049	6.6248
PS	3.716/4.514	1.7/6.02	42.547	6.298/77.16	9.345/2.907	1.2161
				6.309/77.43	9.313/2.896	1.2071
				6.5/82.77	8.878/2.742	1.0682
				6.184/74.08	9.697/3.031	1.3206
	Controller component values					
	$R_{1}$/$R_{2}$	$R_{3}/R_{4}/R_{5}$		$R_{1}$/$R_{2}$	$R_{3}/R_{4}/R_{5}$	
GA	0.13/62.01	412.364/10.19/412.405		0.52/63.05	27.491/10.38/27.534	
	0.13/62.01	418.551/10.34/418.594		1.26/65.07	4.439/9.81/4.481	
	0.13/62	425.389/10.18/425.432		3.29/70.62	0.585/8.52/0.626	
	0.13/62	425.287/10.18/425.329		2.29/67.87	1.298/9.28/1.339	
	0.13/62.01	418.596/10.34/418.638		0.49/62.98	31.156/10.66/31.198	
	0.13/62.01	417.775/10.32/417.818		1.06/64.54	6.582/10.41/6.625	
PS	0.13/62	425.426/10.18/425.468		2.44/68.29	1.174/9.53/1.216	
				2.45/68.31	1.165/9.52/1.207	
				2.57/68.65	1.027/9.24/1.068	
				2.35/68.04	1.279/9.63/1.321	

^1^ × 10^4^. ^2^ × 10^7^. ^3^ × 10^11^. ^4^ × 10^5^. ^5^ × 10^10^. ^6^ × 10^3^.

**Table 6:** Optimized parameters and component values from minimizing criteria (9), (10) and phase margin $\phi_{d}$ when a classical PD controller is being used.

	$J_{1}$		$J_{2}$	
	Optimization results			
	$k_{p}$	$T_{d}$	$k_{p}$	$T_{d}$
GA	9.9988	2.98 $\times \;10^{-6}$	9.9993	2.85 $\times \;10^{-6}$
	9.9983	2.98 $\times \;10^{-6}$	9.999	2.95 $\times \;10^{-6}$
PS	10	2.99 $\times \;10^{-6}$	10	2.95 $\times \;10^{-6}$
	10	2.98 $\times \;10^{-6}$	10	2.97 $\times \;10^{-6}$
	Controller component values			
	$R_{i}$/$R_{f}$	$C_{i}/R_{f}$	$R_{i}$/$R_{f}$	$C_{i}/R_{f}$
GA	1 kΩ/9.9 kΩ	1 μF/2.98 Ω	1 kΩ/9.9 kΩ	1 μF/2.85 Ω
	1 kΩ/9.9 kΩ	1 μF/2.98 Ω	1 kΩ/9.9 kΩ	1 μF/2.95 Ω
PS	1 kΩ/10 kΩ	1 μF/2.99 Ω	1 kΩ/10 kΩ	1 μF/2.95 Ω
	1 kΩ/10 kΩ	1 μF/2.98 Ω	1 kΩ/10 kΩ	1 μF/2.97 Ω

**Table 7:** Performance parameters for both step responses from Figure 7a and Figure 12a, where FOPD is the fractional-order PD controller.

Parameter	Symbol	FOPD	PD
Rise time	$t_{r}$	8.72 μs	9.83 μs
Settling time	$t_{s}$	76.66 μs	144.01 μs
Peak time	$t_{p}$	22.33 μs	24.78 μs
Overshoot	% M_p_	39.6%	51.7%

## Data Availability

Not applicable.

## References

[1] Chandrakasan A.P., Verma N., Daly D.C. (2008). Ultralow-power electronics for biomedical applications. Annu. Rev. Biomed. Eng..

[2] Co M.L., Khouzam J.P., Pour-Ghaz I., Minhas S., Basu-Ray I. (2021). Emerging technologies in cardiac pacing from leadless pacers to stem cells. Curr. Probl. Cardiol..

[3] Azimi S., Golabchi A., Nekookar A., Rabbani S., Amiri M.H., Asadi K., Abolhasani M.M. (2021). Self-powered cardiac pacemaker by piezoelectric polymer nanogenerator implant. Nano Energy.

[4] Adelstein E., Zhang L., Nazeer H., Loka A., Steckman D. (2021). Increased incidence of electrical abnormalities in a pacemaker lead family. J. Cardiovasc. Electrophysiol..

[5] Amara Y., Tebri Z., Larabi Z. (2022). Design, Control, Management, and Performance Analysis of PV-Battery Supercapacitor DC-System Using Buck Converter. Modeling and Control of Static Converters for Hybrid Storage Systems.

[6] Awada E., Radwan E., Nour M. (2022). Robust sliding mode controller for buck DC converter in off-grid applications. Bulletin Electr. Eng. Inform..

[7] Abdurraqeeb A.M., Al-Shamma’a A.A., Alkuhayli A., Noman A.M., Addoweesh K.E. (2022). RST Digital Robust Control for DC/DC Buck Converter Feeding Constant Power Load. Mathematics.

[8] Alarcón-Carbajal M.A., Carvajal-Rubio J.E., Sánchez-Torres J.D., Castro-Palazuelos D.E., Rubio-Astorga G.J. (2022). An Output Feedback Discrete-Time Controller for the DC-DC Buck Converter. Energies.

[9] Sanchez R.O., Rumbo Morales J.Y., Ortiz Torres G., Pérez Vidal A.F., Valdez Resendiz J.E., Sorcia Vázquez F.d.J., Nava N.V. (2022). Discrete State-Feedback Control Design with D-Stability and Genetic Algorithm for LED Driver Using a Buck Converter. Int. Trans. Electr. Energy Syst..

[10] Nizami T.K., Chakravarty A., Mahanta C., Iqbal A., Hosseinpour A. (2022). Enhanced dynamic performance in DC–DC converter-PMDC motor combination through an intelligent non-linear adaptive control scheme. IET Power Electron..

[11] Devaraj S.V., Gunasekaran M., Sundaram E., Venugopal M., Chenniappan S., Almakhles D.J., Subramaniam U., Bhaskar M.S. (2021). Robust Queen Bee Assisted Genetic Algorithm (QBGA) Optimized Fractional Order PID (FOPID) Controller for Not Necessarily Minimum Phase Power Converters. IEEE Access.

[12] Mollaee H., Ghamari S.M., Saadat S.A., Wheeler P. (2021). A novel adaptive cascade controller design on a buck–boost DC–DC converter with a fractional-order PID voltage controller and a self-tuning regulator adaptive current controller. IET Power Electron..

[13] Aseem K., Kumar S.S. (2021). Hybrid k-means Grasshopper Optimization Algorithm based FOPID controller with feed forward DC–DC converter for solar-wind generating system. J. Ambient Intell. Humaniz. Comput..

[14] Tepljakov A., Alagoz B.B., Yeroglu C., Gonzalez E.A., Hosseinnia S.H., Petlenkov E., Ates A., Cech M. (2021). Towards Industrialization of FOPID Controllers: A Survey on Milestones of Fractional-Order Control and Pathways for Future Developments. IEEE Access.

[15] Ghamari S.M., Narm H.G., Mollaee H. (2022). Fractional-order fuzzy PID controller design on buck converter with antlion optimization algorithm. IET Control Theory Appl..

[16] Karad S.G., Thakur R. (2022). Fractional order controller based maximum power point tracking controller for wind turbine system. Int. J. Electron..

[17] Paul R. (2022). Fractional Order Modified AWPI Based DC-DC Converter Controlled SEDC Motor. International Conference on Computational Techniques and Applications.

[18] Saleem O., Shami U.T., Mahmood-ul Hasan K. (2019). Time-optimal control of DC-DC buck converter using single-input fuzzy augmented fractional-order PI controller. Int. Trans. Electr. Energy Syst..

[19] Izci D., Hekimoğlu B., Ekinci S. (2022). A new artificial ecosystem-based optimization integrated with Nelder-Mead method for PID controller design of buck converter. Alexandria Eng. J..

[20] Izci D., Ekinci S. (2022). A novel improved version of hunger games search algorithm for function optimization and efficient controller design of buck converter system. e-Prime Adv. Electr. Eng. Electron. Energy.

[21] Izci D., Ekinci S., Hekimoğlu B. (2022). Fractional-order PID controller design for buck converter system via hybrid Lévy flight distribution and simulated annealing algorithm. Arab. J. Sci. Eng..

[22] Mohadeszadeh M., Pariz N., Ramezani-al M.R. (2022). A fractional reset control scheme for a DC-DC buck converter. Int. J. Dyn. Control.

[23] Jia Z., Liu L., Liu C. (2022). Dynamic Analysis and Fractional-Order Terminal Sliding Mode Control of a Fractional-Order Buck Converter Operating in Discontinuous Conduction Mode. Int. J. Bifurc. Chaos.

[24] Sorouri H., Sedighizadeh M., Oshnoei A., Khezri R. (2022). An intelligent adaptive control of DC–DC power buck converters. Int. J. Electr. Power Energy Syst..

[25] Cengelci E., Garip M., Elwakil A.S. (2022). Fractional-order controllers for switching DC/DC converters using the K-factor method: Analysis and circuit realization. Int. J. Circuit Theory Appl..

[26] Nirmala R.J., Balachandran K., Trujillo J.J. (2017). Null controllability of fractional dynamical systems with constrained control. Fract. Calc. Appl. Anal..

[27] Yaghooti B., Hosseinzadeh M., Sinopoli B. Constrained control of semilinear fractional-order systems: Application in drug delivery systems. Proceedings of the 2020 IEEE Conference on Control Technology and Applications (CCTA).

[28] Sopasakis P., Sarimveis H. (2017). Stabilising model predictive control for discrete-time fractional-order systems. Automatica.

[29] De Oliveira E.C., Tenreiro Machado J.A. (2014). A review of definitions for fractional derivatives and integral. Math. Probl. Eng..

[30] Carlson G., Halijak C. (1964). Approximation of fractional capacitors (1/s)ˆ(1/n) by a regular Newton process. IEEE Trans. Circuits Theory.

[31] Oustaloup A., Levron F., Mathieu B., Nanot F.M. (2000). Frequency-band complex noninteger differentiator: Characterization and synthesis. IEEE Trans. Circuits Syst.-I Fundam. Theory Appl..

[32] Monje C.A., Chen Y., Vinagre B.M., Xue D., Feliu-Batlle V. (2010). Fractional-Order Systems and Controls: Fundamentals and Applications.

[33] Charef A., Sun H., Tsao Y., Onaral B. (1992). Fractal system as represented by singularity function. IEEE Trans. Autom. Control.

[34] El-Khazali R. (2013). Fractional–order PI^λ^D^μ^ controller design. Comput. Math. Appl..

[35] El-Khazali R. (2015). On the biquadratic approximation of fractional–order Laplacian operators. Analog Integr. Circuits Signal Process..

[36] Erickson R.W., Maksimovic D. (2007). Fundamentals of Power Electronics.

[37] Rana N., Latiff M.S.A., Abdulhamid S.M., Chiroma H. (2020). Whale optimization algorithm: A systematic review of contemporary applications, modifications and developments. Neural Comput. Appl..

[38] Mirjalili S., Lewis A. (2016). The whale optimization algorithm. Adv. Eng. Softw..

[39] Åström K.J., Murray R.M. (2021). Feedback Systems: An Introduction for Scientists and Engineers.

[40] Kochenderfer M.J., Wheeler T.A. (2019). Algorithms for Optimization.

